# Shared neural correlates for building phrases in signed and spoken language

**DOI:** 10.1038/s41598-018-23915-0

**Published:** 2018-04-03

**Authors:** Esti Blanco-Elorrieta, Itamar Kastner, Karen Emmorey, Liina Pylkkänen

**Affiliations:** 1 Department of Psychology, New York University, New York, NY USA; 2 NYUAD Institute, New York University Abu Dhabi, Abu Dhabi, UAE; 3 Department of Linguistics, New York University, New York, NY USA; 4 Institut für Anglistik und Amerikanistik, Humboldt-Universität zu Berlin, Berlin, Germany; 5 School of Speech, Language and Hearing Sciences, San Diego State University, San Diego, CA USA

## Abstract

Research on the mental representation of human language has convincingly shown that sign languages are structured similarly to spoken languages. However, whether the same neurobiology underlies the online construction of complex linguistic structures in sign and speech remains unknown. To investigate this question with maximally controlled stimuli, we studied the production of minimal two-word phrases in sign and speech. Signers and speakers viewed the same pictures during magnetoencephalography recording and named them with semantically identical expressions. For both signers and speakers, phrase building engaged left anterior temporal and ventromedial cortices with similar timing, despite different linguistic articulators. Thus the neurobiological similarity of sign and speech goes beyond gross measures such as lateralization: the same fronto-temporal network achieves the planning of structured linguistic expressions.

## Introduction

Most human languages are expressed via gestures of the vocal tract, that is, as speech. However, the existence of sign languages shows that speech is not a necessary modality for language: the meanings and complex structures of language can also be coded as manual gestures. Research on the architecture of sign languages has shown that they share a lot of structural features with spoken languages^1^, but much less is known about the neurobiological basis of this similarity. At a gross level, the neural implementation of both sign and speech relies largely on the left hemisphere^2,3^. We also have evidence that the general networks recruited for the production of single words^4^ and of narratives^5^ exhibit overlap for sign and speech. Recent work also indicates that comprehending both signs and words activates overlapping left frontotemporal regions during lexical-semantic processing (~300 ms), while early sensory processing (~100 ms) is confined to modality specific regions^6^ (see also^7^). However, whether the specific linguistic computations that make up these complex tasks are neurobiologically similar between the two types of languages remains unknown (particularly for language planning and production).

Since signed and spoken languages are perceptually and articulatorily distinct, their neural bases must diverge in many ways: the programming of a manual gesture is clearly different from movements of the mouth, and the perception of sign language engages the visual cortex while speech is processed in the auditory cortex. Thus the neurobiological similarity between the two types of languages would have to lie at a more abstract level, i.e., in the structural and semantic properties of language, which, by hypothesis, are encoded in an amodal representational format within the brain. However, discovering such abstract similarity in the face of abundant lower-level differences is methodologically non-trivial.

**Figure 1:**
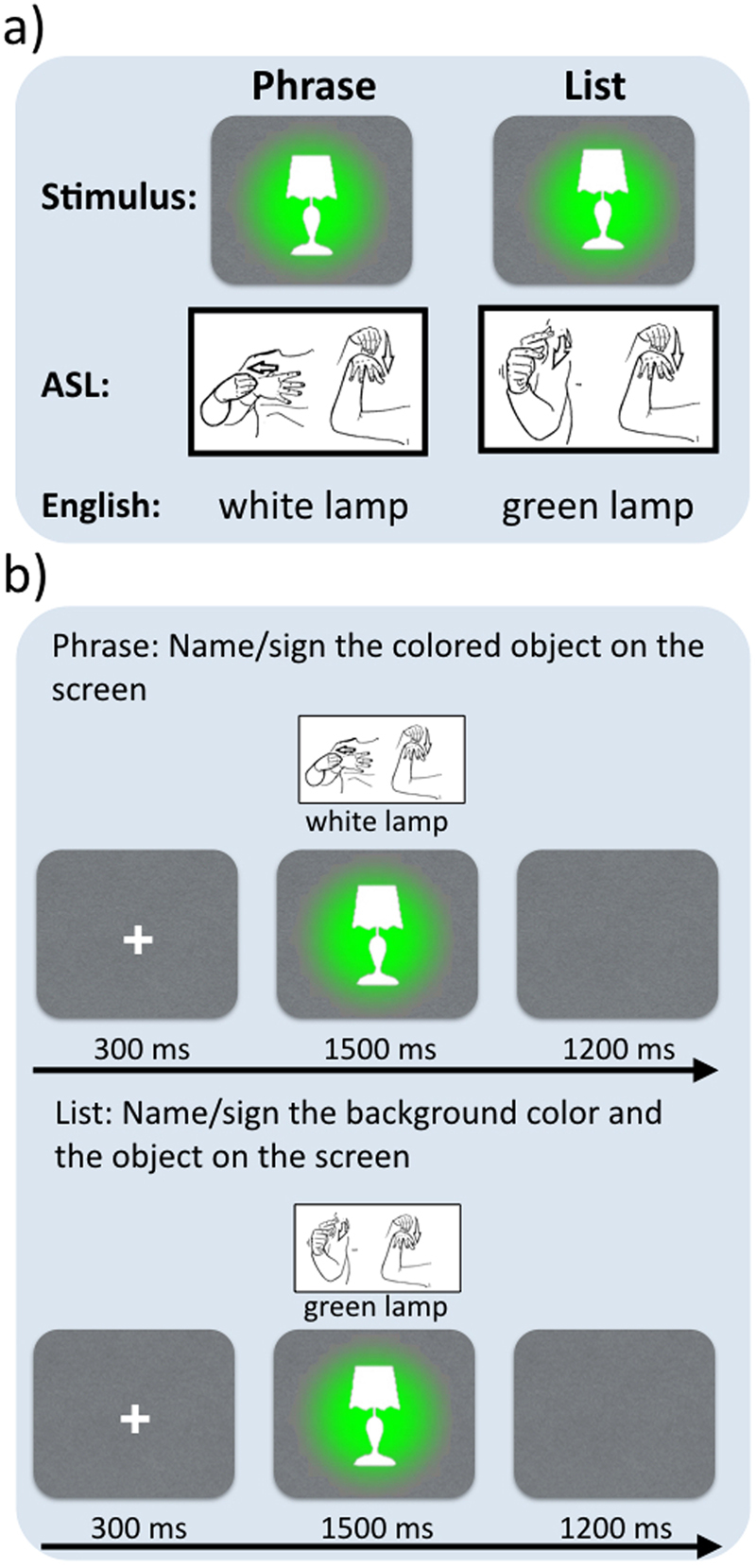
(**a**) Stimulus design with example productions for ASL and English. (**b**) Trial structure for the Phrase and List tasks. (Pictures of ASL signs are courtesy of Dr. Bill Vicars and www.lifeprint.com. Used by permission).

At the heart of humans’ abstract linguistic capacity lies our ability to combine words into larger expressions – this is what gives language its infinite power to convey novel thought. Thus, if signed and spoken languages are fundamentally expressions of the same underlying system, they should share at least some combinatory processes. We focused on this question in language production, which, unlike comprehension, allowed us to control the perceptual properties of the stimuli eliciting combinatory processing. Specifically, we presented identical pictures of colored objects to both hearing speakers of English and Deaf signers of American Sign Language (ASL) and asked them to describe the pictures with combinations of color-describing adjectives (e.g., *white*) and object-describing nouns (e.g., *lamp*). Crucially, each picture contained two colors: the color of the object and the color of the background, as shown in Fig. 1. In one task, we asked our participants to describe the color of the object and then the shape of the object, resulting in noun phrases such as *white lamp*, *green bell*, etc. These utterances all engaged the combinatory rules that build noun phrases in English and in ASL; our aim was to discover the degree of neural overlap between the associated brain activities in the two languages. The control task involved the same pictures but now the participants were asked to describe the color of the background followed by the object-name. While this resulted in sequences of adjectives and nouns that were lexically identical to the phrase production task (across the full stimulus set), the adjectives did not provide features for the objects described by the nouns and thus the two words did not syntactically make up a phrase. Instead, they formed a list of two words that simply happened to belong to the categories adjective and noun. This design allowed us to control not only the perceptual properties of the stimuli, which were identical for the phrase and list conditions, but also of the actual words/signs uttered, which were identical for the two conditions. In this way, we were able to isolate the effects associated with the combinatory rule that composes adjectives and nouns into noun phrases and compare the neural effects of this rule between ASL and English.

To capture the brain activity associated with the structural planning of the phrases, known to occur within a few hundred milliseconds after the onset of the picture^8^, the behavioral task was accompanied by magnetoencephalography (MEG), which unlike any other noninvasive neuroimaging technique, could provide us with both a millisecond temporal resolution and relatively good spatial localization of the neural currents generating the recorded signal. The localized electromagnetic correlates of basic phrase building have been previously characterized for spoken language, both in comprehension^9^ and production^8^. Thus, we were able to directly focus on testing the involvement of these processes in sign language. Specifically, when English speakers plan minimal two word phrases, neural sensitivity to combinatorial requirements in the planned utterance begins in ventromedial prefrontal cortex (vmPFC) at 150–200 ms, followed by increased activity in the left anterior temporal lobe (LATL) shortly afterwards^8^. To test for the involvement of the vmPFC and LATL in phrase building across sign and speech, these areas were treated as regions of interest in our analysis. In addition, we analyzed two control regions, the angular gyrus (AG) and the left inferior frontal gyrus (LIFG), neither of which have shown sensitivity to simple composition in production but have been implicated for other aspects of integrative processing in comprehension^10–12^. The time course for the involvement of these regions was compared across our stimulus and language manipulations millisecond by millisecond.

Additionally, to find in multivariate space whether the patterns of composition were indeed similar across languages, we ran a representational similarity analysis (RSA), in which we tested for a correlation between the actual data and a model in which the contrast between the phrase and list stimuli was identical for the two languages. This analysis sought to confirm our univariate results and additionally sharpen the level of precision of the analysis: in addition to testing the quantitative difference in the amount of activity elicited by each condition, we additionally tested whether English and ASL phrases evoke the same patterns of activity across individual stimuli.

It is important to note that although our design achieved an unusually clean contrast between phrasal and non-phrasal productions, since we fully controlled both the visual stimulus and the produced words, our different trial types did require differential inhibitory processing. In particular, the List trials required somewhat unnatural naming and consequently felt intuitively harder. This is presumably due to the need to inhibit the object-color on these trials, i.e., the concept one would naturally place in the prenominal modifier position. Given that more effortful cognitive control typically recruits more neural activity^13,14^, this factor therefore biased our design against a replication of our prior results, i.e., increased activation for the phrasal productions as compared to lists. At the same time though, it also made a potential replication particularly compelling, since the replication would show that the combinatory effects are strong enough to override this counteracting inhibitory force.

Finally, we would also like to note that possible main effects of language were confounded by the fact that the English experiment was conducted at NYU Abu Dhabi and the ASL experiment at NYU New York. For this reason, we will refrain from deriving any conclusions from main effects of language.

## Results

### Behavioral data

**Figure 2:**
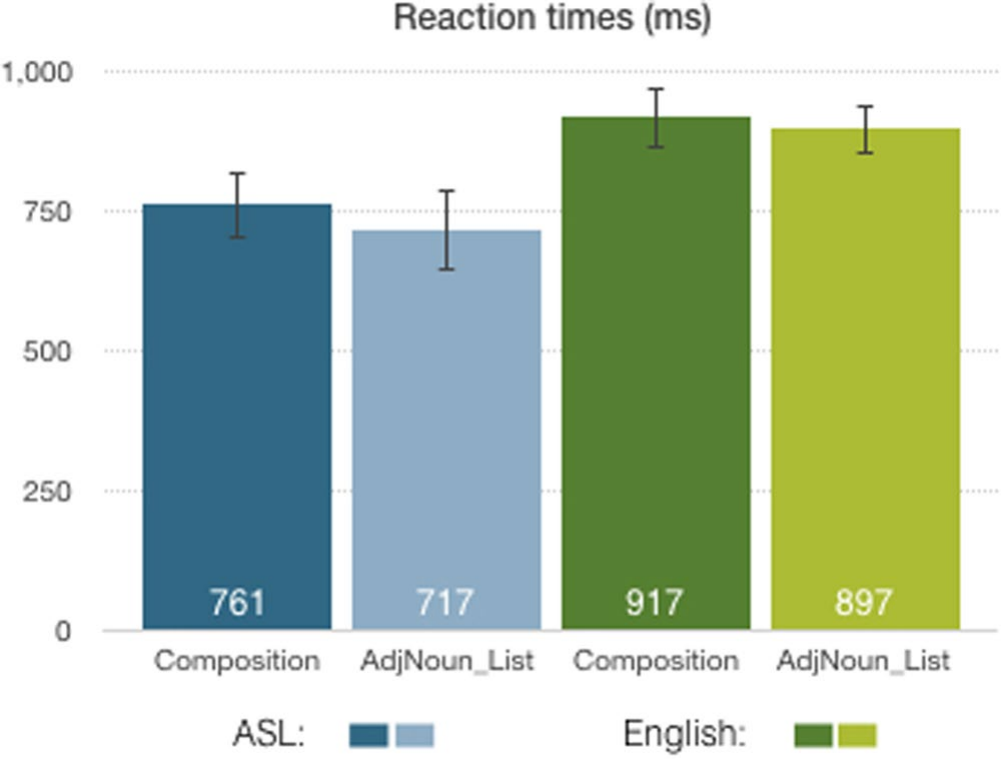
Reaction time results for ASL and English (error bars show SEM).

Behaviorally, the compositionality of the phrase did not produce reliable reaction time differences, although phrase productions elicited slightly slower speech/sign onsets than list productions (761 ms vs. 717 ms in ASL and 917 ms vs. 897 ms in English; (*F*(1,18) = 0.56; *p* = 0.461; see Fig. 2). There was a main effect of language, since utterance onset was faster for sign than for speech (*F*(1,18) = 4.877; *p* = 0.042). Accuracy was at ceiling for both English speakers and signers (above 97% for both groups).

### MEG data

**Figure 3:**
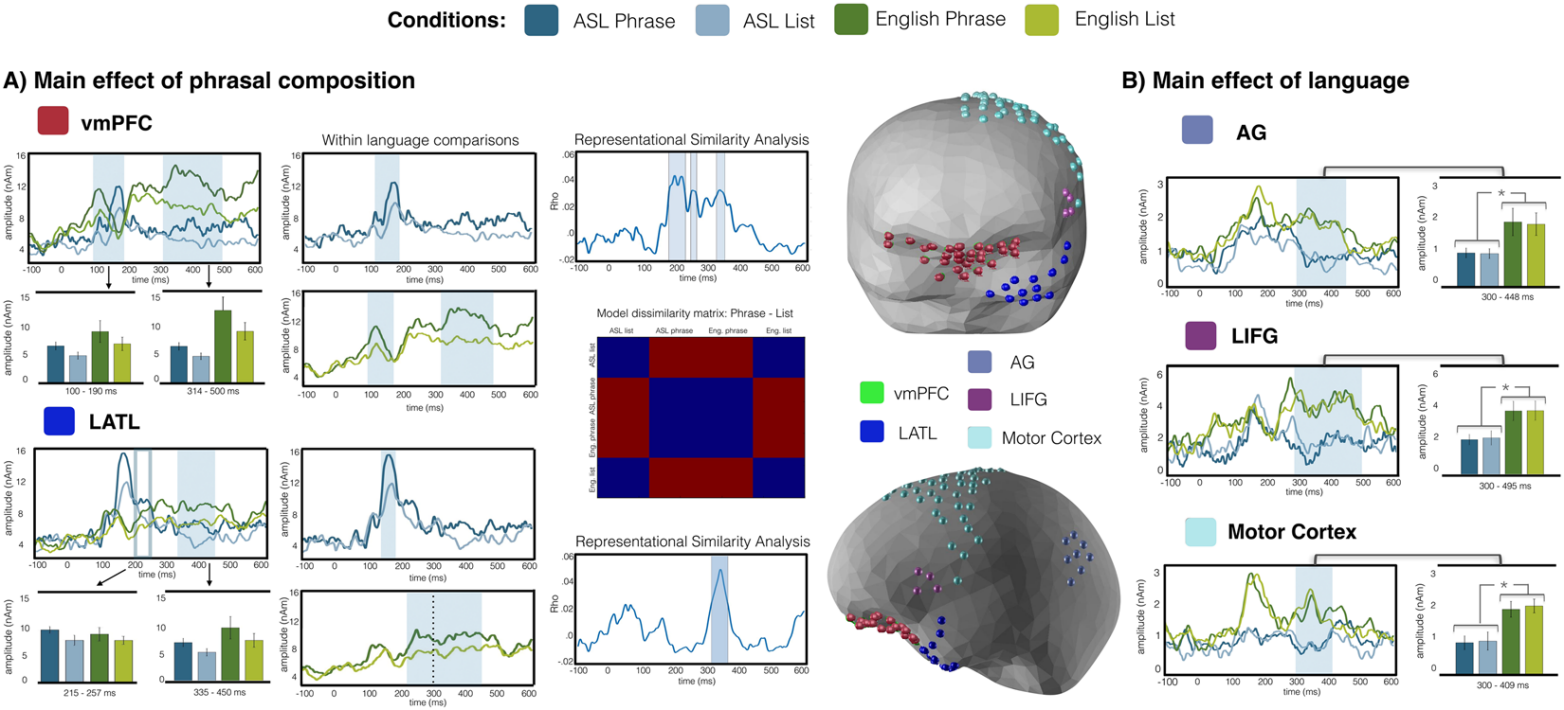
MEG results for the left lateral ROIs and the vmPFC. The sources included in each ROI are displayed in the middle on an average BESA brain. Panel A shows (i) effects of phrasal composition in the vmPFC and LATL in both languages, with phrases eliciting higher activation than lists and (ii) results for the RSA analysis in the same regions, with brain activity tested against a model that coded trials as similar in terms of their compositionality (or lack thereof) irrespective of language. Panel B displays the additional regions (AG, LIFG and motor cortex) showing only main effects of language. Shading on waveforms stands for p < 0.05 and boxing for p < 0.1, corrected with non-parametric permutation tests (Maris & Oostenveld, 2007). (vmPFC = ventromedial prefrontal cortex; LATL = left anterior temporal lobe; AG = angular gyrus; LIFG = left inferior frontal gyrus).

**Figure 4:**
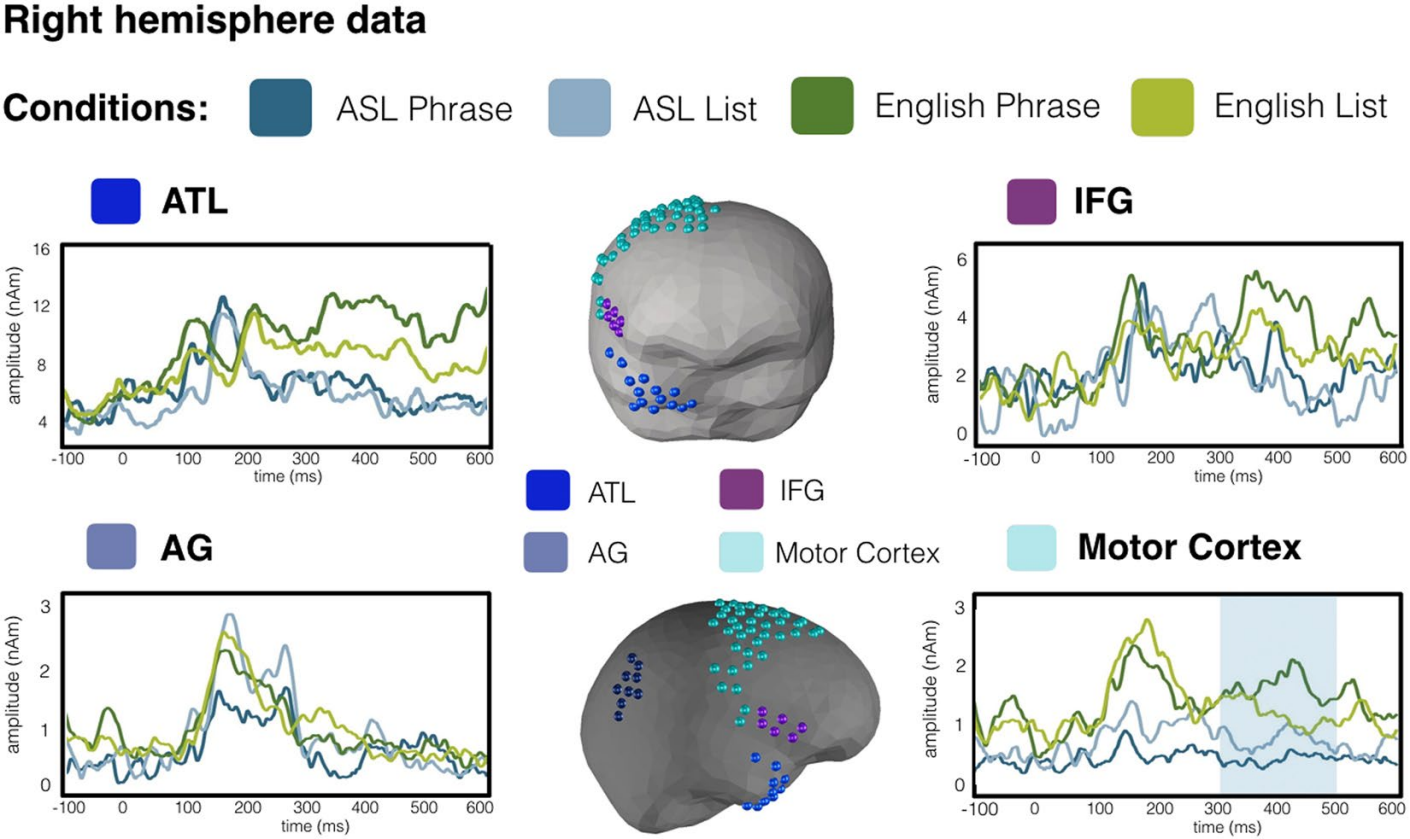
MEG results for regions of the right hemisphere. The phrase vs. list contrast did not affect the right lateral ROIs. Instead only a main effect of Language was observed in the motor cortex. Shading stands for p ≤ 0.05, corrected.

Given the millisecond temporal resolution of MEG, we were able to assess not only if similar regions were sensitive to the presence of phrasal structure in the planned utterances, but also whether the time-course of such effects was shared between sign and speech. If the structural planning of phrases involves a universal, amodal combinatory mechanism, the phrasal effects observed for sign and speech should overlap not only in space but also in time. This is exactly what we observed (Fig. 3A): in the LATL and the vmPFC, a two-phase activity increase was elicited for phrases over lists in both language modalities, with a slightly earlier time-course for the vmPFC than the LATL, consistent with prior reports for speech^8^. Specifically, the vmPFC showed a main effect of phrasal composition at 100–190 ms (*p* = 0.033) and then again at 314–500 ms (*p* < 0.001), with the LATL showing a similar profile slightly later, at 215–257 (*p* = 0.091) and at 335–450 ms (*p* = 0.030). These main effects of phrase composition were not qualified by interactions with language, as one would expect if there would indeed be a dissimilarity in the combinatorial processes in either region for sign vs. speech. The pairwise comparisons within language showed an early vmPFC effect both in ASL (*p* = 0.013, 119–191 ms) and English (p = 0.038, 100–162 ms) while the later vmPFC effect only reached significance in English (*p* = 0.001, 313–497 ms; ASL *p* = 0.11, 320–500; Fig. 3A). In the LATL, patterns were again similar: the early increase reached significance in ASL (*p* = 0.044, 145–189 ms) and English (*p* = 0.026, 221–300 ms), and the later one only in English (*p* = 0.027, 300–458 ms). Finally, no effects of phrasal structure were observed in the AG or the LIFG (all clusters *p* > 0.5). Hence, although there is a slight variation in the timing of the effects, possibly due to the fact that the timecourse of the planning production is not exactly parallel in both domains, the morphology and overall timing of the effects across languages are remarkably similar. The analysis in the right hemisphere revealed no effects of the Phrase vs. List manipulation (Fig. 4). Effects of Language were not analysed as they would have been confounded by the use of different MEG instruments for each language.

Additionally, to further assess the similarity of the computations in these regions, we ran a representational similarity analysis (RSA^15^) in the regions showing similar combinatory profiles in ASL and English in the main univariate analysis. In other words, we aimed to find in multivariate space whether the patterns of composition were indeed similar across languages. In order to do this, we designed a model representational dissimilarity matrix that coded trials as similar in terms of their compositionality (or lack thereof) irrespective of language, and tested this model against brain activity. This analysis revealed that in time-windows overlapping with the significant main effects in the univariate ROI analysis, LATL and vmPFC time-courses were reliably similar to a model with no difference between ASL and English as regards the list vs. phrase contrast (Fig. 3A). Specifically, in the LATL, similarity between the model and the data was significant between 310–357 ms (cf., main effect at 335–450 ms in ANOVA) and in the vmPFC, at 187–235 ms, 250–267 ms and 324–348 ms (cf., sig. main effects in ANOVA at 100–190 ms and 314–500 ms). Like in the univariate analysis, evidence for language-insensitive phrasal effects onset earlier in the vmPFC than in the LATL.

## Discussion

In this work, we asked if the planning of phrases during language production engages the same neural circuitry in speech and sign. The odds were stacked against any shared effect, as our English and ASL experiments involved not only different languages, but also different articulators, experimental participants, the MEG instrument used for the neural recordings, and every aspect of the environment (with ASL signers residing in and around New York and English speakers living in Abu Dhabi). Nevertheless, the presence of phrasal structure in a planned two-word utterance elicited spatio-temporally overlapping effects in sign and speech. Importantly, this overlapping profile reflected activity increases for phrasal structures over lists. This pattern alleviated concerns that the differences observed in this study derived solely from differences in generic inhibitory control requirements: Since previous research^16–19^ has shown that increased inhibitory demands elicit increased activity, if the shared effect that we observed would indeed be just a result of different inhibitory demands across conditions, we should have observed increased activity for Lists over Phrases. However, we found the opposite pattern.

The combinatory effects were centered in two anterior regions as predicted by similar - though not as tightly controlled - manipulations in prior studies on English^8,20^. Since the present design had no visual or lexical confounds, the observed effects most likely reflected the distinct combinatorial properties of the two types of expressions. The similarity of the effects was also supported by a representational similarity analysis (RSA), testing for a correlation between the actual data and a model in which the contrast between the phrase and list stimuli was identical for the two languages. These findings add an important new dimension to our understanding of the neurobiology of sign production, which has so far primarily focussed either on single signs^21–23^ or on aspects of sign languages that are not shared with speech such as bimanual articulation or the use of signing space^24,25^.

The loci of our combinatory effects, the left anterior temporal lobe and the ventromedial prefrontal cortex, matches the network of semantic hubs that robustly emerges from a large body of hemodynamic work on conceptual and combinatory processing^26^. While we also analysed other regions that likely play a role in the combinatory processing of language, such as the angular gyrus – a strong “conceptual hub”^26^ – and the left inferior frontal gyrus, another potentially integrative site on the basis of hemodynamic measurements (e.g,^12,27^), we only detected effects of composition in the LATL and vmPFC, consistent with prior MEG results^8,9^. Instead of basic composition, MEG measurements localized in the AG have shown sensitivity to the number of relations encoded by a concept^28^, while the LIFG has been modulated by the presence of long-distance dependencies^29^. Both of these findings are consistent with sizeable hemodynamic literatures (AG^30,31^; LIFG:^32^), suggesting that these regions are involved in sentence processing but not in the lowest level of composition per se.

In all, our findings suggest that within a combinatory network that undeniably extends beyond the LATL and vmPFC, these two hubs house at least some of the most elementary combinatory routines that subserve the construction of phrases, with other regions potentially contributing to different computations. More specifically, a series of recent studies has implicated the LATL for the conceptual aspects of combination, as opposed to syntactic or semantic composition more generally^33,34^). For example, in production, combinatory effects in the LATL are not observed for quantificational phrases such as *two cups*, in which the number word does not add a conceptual feature to the noun but instead enumerates the number of tokens belonging to the category described by the noun^20^. Further evidence from comprehension suggests that the function of the LATL may be limited to relatively simple cases of conceptual combination, perhaps mainly computing the intersection of features^35,36^. The vmPFC also seems to have a clearly semantic role, but contrary to the LATL, it robustly responds to complex cases of semantic composition such as so-called coercion constructions, involving a syntax-semantics mismatch that has to be resolved via a special rule^37–39^. In comprehension, vmPFC activity peaks relatively late, at around 400 ms, whereas in production, it onsets early, at ~150–200 ms. This finding is consistent with the hypothesis that this activity represents a message level interpretation that constitutes the starting point for production processes and the output of combinatory processing in comprehension.

Given the millisecond temporal resolution of MEG, we were able to assess not only the spatial, but also the temporal extent of any shared effects between ASL and English. Notably, our main effects indicated a bi-phasic temporal profile in the phrasal effects, consistent with prior findings for English^8^. This pattern likely reflects multiple stages of combinatory processing, including both the construction of a combinatory message (say, the combined concept of a white lamp) as well as the planning of the specific phrase to express that message. In light of the literature discussed above, the former could plausibly be a vmPFC-mediated process and the latter an LATL-mediated one. Further, models of language production have hypothesized that production incorporates comprehension processes in the form of speakers monitoring their own (planned or already produced) utterances^40^ (in other words, after constructing the to-be-uttered utterance, the producer is thought to comprehend it internally. Internal monitoring also occurs for sign language^41^. Since the LATL and vmPFC have also been shown to be engaged for phrase comprehension^9^, as one would expect if their role is modality general, it is possible that the late vmPFC and LATL effects, centered around 400 ms after picture onset, could reflect comprehension of a previously constructed combinatory plan. As regards convergence between our uni- and multivariate analyses, the clearest consistency was observed in the later time-window, exhibiting both reliable main effects of phrasal structure in the LATL and vmPFC as well as significant correlations between the actual data and our model in the RSA analysis, which assumed no representational differences between the languages.

When evaluating the strength of the evidence presented here, an additional important consideration is the inherent limitations of the employed imaging technique. While MEG has exquisite temporal resolution, its spatial resolution is fuzzier. Thus on the basis of the current findings one cannot rule out the possibility that phrasal composition in sign and speech might recruit nearby but distinct neural populations (just like on the basis of hemodynamic data one cannot rule out that a similarity localized effect occurs at different times for different conditions). This limitation is compounded by the fact that the regions in question, left anterior temporal lobe and ventromedial prefrontal cortex, are known to be activated by many different types of tasks. Thus while our findings clearly conform to the hypothesis that ASL and English engage similar combinatory computations, they are also consistent with any hypothesis in which there is some difference between these computations that cannot be detected with the methods we used. Despite this unavoidable limitation, the present study offers a characterization of basic combinatory processes in sign and speech at a resolution not available in prior literature, both in terms of the measurement technique and in terms of the linguistic process measured.

In conclusion, we have characterized the spatiotemporal dynamics of the planning of phrases in ASL and English, offering the first detailed study of a specific – albeit still multistage – computation across these two very different language types. The similar recruitment of left anterior and medial prefrontal cortices suggests a deep anchoring of combinatory processing in these two integrative hubs and offers further evidence for common neural basis for sign and speech. Thus our findings offer a starting point for a more complete neurobiological characterization of the interleaved lexical and compositional processes that together orchestrate language production.

## Methods

### Participants

In order to investigate the similarities between ASL and English, one could independently test a group of native speakers of each language or alternatively, a population that is bilingual and fluent in both languages (i.e., CODAs). Hearing bilinguals who speak English and sign ASL are almost always English dominant (even if they learned ASL from birth) because English is their schooling language and the language of commerce and everyday interactions. For this reason, we decided in favor of testing a group of English monolinguals and a group of deaf ASL signers. Deaf signers in this study do not frequently produce speech, cannot hear English, and use ASL as their primary, everyday language. So although they may read English, they do not use spoken English, making the two groups as monolingual as the circumstances allow. This enabled us to maximally avoid cross language confounding effects. Based on^8^, which established the current paradigm and the expected effect size, we aimed for a minimum of 20 participants for the entire study (in the prior study, Exp. 1 had 20 participants and Exp. 2 14 participants). This somewhat modest target took into account the difficulty in recruiting right-handed deaf native ASL signers with no cochlear implants or contraindications for undergoing MEG (e.g., no problematic fillings, permanent retainers or tattoos) and the fact that we were looking for an established effect. Over a period of 22 months, 15 ASL signers participated in the study; of these, we were able to include 11 in the data analysis (as explained below). The total number of English participants was 19, but to equate the sample size, we only report results from the first 11. The results for the English experiment at *n* = 11 and *n* = 19 did not qualitatively differ, and we include the results for *n* = 19 in the additional materials. In sum, 22 participants are included in the analyses reported below. All experimental protocols were approved by New York University Institutional Review Board and conducted in accordance with the relevant guidelines and regulations, and all participants signed an informed consent form before taking part in the experiment.

### English

The 19 English speakers were all right-handed and monolingual native speakers of English from the United Kingdom or the United States and did not report knowledge of any other language beyond English (9 female, 10 male (ages: *M* = 25.6, *SD* = 7.3). All were neurologically intact, with normal or corrected-to-normal vision and all provided informed written consent. As explained above, to enable comparisons between the ASL and the English experiments, we matched the sample sizes of both experiments to the lowest sample size (ASL experiment, *n* = 11). In order to do this, data for the last 8 English participants were not included in the analyses reported in here (results for the full data set of 19 participants are presented in the Supplementary figure). Of the eleven English speakers included in the analysis, 5 were female and 6 male (ages: *M* = 26, *SD* = 8.87).

### ASL

The 15 ASL signers were also all right-handed and neurologically intact, with normal or corrected-to-normal vision (7 female, 8 male (ages: *M* = 38.06, *SD* = 7.43). All provided informed written consent. All participants were native deaf signers who learned ASL from signing family members in infancy (*M*_*age of acquisition*_ = 1.4, *SD*_*age of acquisition*_ = 0.92). None of our participants reported knowledge of any other sign language beyond ASL. We removed from the final analysis three participants due to excessive noise in their recordings, and one additional participant who reported to be left-handed. Of the eleven ASL signers included in the analysis, 6 were female and 5 male (ages: *M* = 37.5, *SD* = 7.16).

### Stimuli and experimental design

The experiment consisted of 200 trials in which participants were presented with a colored picture on a colored background (e.g., a red plane on a green background) and were asked to name it (in the English experiment) or sign it (in the ASL experiment). The stimuli were kept constant across all conditions, assuring that there was no perceptual variation amongst them. In the Phrase condition, participants were required to describe the picture on the screen by naming its color and shape (e.g., *red bag*). In the List condition, participants were required to name the color of the background and the shape of the object in an enumerative fashion (e.g., *green*, *bag*) (Fig. 1A). In one experiment, hearing participants performed the tasks with overt speech, and in a second experiment, deaf participants performed the tasks in ASL.

The colored objects were 25 unique combinations created from a subset of five object shapes (bag, bell, cane, lamp, plane) and five colors (black, brown, green, red, white). Each of these combinations was assigned a background color in a counterbalanced fashion, such that all colors were presented the same number of times in the background and were equally distributed across all different shapes. Subsequently, we created the reversed version of these 25 unique combinations (i.e., if one of the combinations was a red plane on a green background, we also created the reverse: a green plane on a red background), yielding 50 stimuli pictures. These 50 items formed the stimuli set that was presented twice in each condition to elicit 100 trials per condition. Items were arranged in blocks of 25 items, resulting in 4 blocks per condition. Items within each block were randomized across participants and blocks belonging to different conditions were randomly interleaved during the experiment. Prior to the beginning of each block, instructions about the upcoming task were presented. Both the ASL and the English experiment recordings included additional experimental conditions of no interest for the current purposes. Because ASL allows both adjective-noun and noun-adjective orders, signers were explicitly instructed to produce the adjective-noun order for the phrase task.

All the selected nouns and adjectives were monosyllabic words in English and short monomorphemic signs in American Sign Language. This control was crucial to avoid differences in reaction times derived from longer motor preparation in word or sign utterances with multiple syllables or long movements. All signs were inherently one-handed. All pictures were presented foveally using Presentation (Neurobehavioral System Inc., California, USA) for the English speakers and Psychtoolbox for the signers, on a screen subtended in a range from 55.16° height and 33.36° width on a screen ~85 cm from the participant.

### Procedure

Before recording, each participant’s head shape was digitized using a Polhemus dual source handheld FastSCAN laser scanner (Polhemus, VT, USA). For the English experiment MEG data were collected in the Neuroscience of Language Lab in NYU Abu Dhabi using a whole-head 208 channel axial gradiometer system (Kanazawa Institute of Technology, Kanazawa, Japan). For the ASL experiment MEG data were collected in the KIT/NYU MEG Lab in NYU New York, using a whole-head 157-channel axial gradiometer system (Kanazawa Institute of Technology, Japan). During both experiments, participants lay in a dimly lit, magnetically shielded room. Vocal responses were captured with an MEG compatible microphone (Shure PG 81, Shure Europe GmbH). Sign initiation was defined as the start of the hand movement, which was captured with a photodiode. At the beginning of all trials, participants rested their signing hand flat on their chest.

In all conditions, trials began with a fixation cross (300 ms), followed by the presentation of the stimuli. The picture remained onscreen until speech onset or until timeout (1500 ms) for English speakers or until timeout for signers. Participants were allowed 1200 ms to finish their response before the fixation cross for the following trial would appear (Fig. 1B). The entire recording lasted ~45 min.

### Data acquisition and preprocessing

MEG data were recorded at 1000 Hz (200 Hz low-pass filter), time locked to the presentation of the stimulus. The epochs were created from 100 ms before to 600 ms after picture onset, and noise was reduced via the Continuously Adjusted Least-Squares Method^42^ in the MEG Laboratory software (Yokogawa Electric Corporation and Eagle Technology Corporation, Tokyo, Japan). For artifact rejection, and to warrant that no motion artifact was present in our data, we followed the same strict artifact rejection routine that we have followed in previous MEG production studies^16–19^: (1) removing all trials that contain naming latencies within our epoch, (2) removing all individual epochs that contain amplitudes >2500 fT/cm for any sensor after noise reduction, (3) visualizing all individual epochs before averaging and rejecting any epoch that contains any sudden increases in the magnitude of the signal caused by artifacts (be it muscular movements or other reasons), and (4) applying a 40 Hz low pass filter that should eliminate any remaining oral movement (gamma-frequency range (>40 Hz) is reportedly the one affected by muscle artifact contamination such as phasic contractions^43,44^). This resulted in the exclusion of 16.73% of the trials in the English experiment (9.97% *sd*), leaving 166.54 trials on average per participant (49.86 *sd*). In the ASL study, 36% of the trials were rejected, leaving 128 trials on average per participant (16.1 *sd*). This resulted in an average of 41.5 trials per condition in English and 32 trials per condition in ASL. Crucially, our design was also based on behavioral evidence that conceptual and grammatical encoding for adjective-noun productions is completed before articulation begins^45,46^. Hence, by measuring activity elicited by the picture, we obtained uncontaminated spatio-temporal maps of activity during combinatorial processing (for further discussion on how the paradigm avoids motion artifact contamination, see^8^).

Data were averaged for each condition and participant. Averages were low-pass filtered at 40 Hz and high-pass filtered at 1 Hz. This strict high pass filter was necessary to analyze data acquired at NYU facilities in lower Manhattan. To keep analysis procedures maximally parallel between the two experiments, the same filter was applied to the English experiment data collected in Abu Dhabi. A 100 ms interval (−100 0 ms) was used to construct the noise covariance matrix and apply a baseline correction. Using the normalized grand average of all trials for a particular participant, the inverse solution was computed from the forward solution. To estimate the distributed electrical current image in the brain at each time sample we used the Minimum Norm Approach^47^ on an average brain as implemented in BESA Research 6.0. The sources were evenly distributed using 1500 standard locations 10% and 30% below the smoothed standard brain surface (750 for each shell). The inverse solution problem was stabilized by the minimum norm mathematical constraint: Out of the many current distributions that could account for the recorded sensor data, the solution with the minimum L2 norm (i.e., the minimum total power of the current distribution) was used. Additionally, we applied depth weighting so that both deep and superficial sources produced a similar, more focal result. The spatio-temporal weighting was conducted to assign large weight to the sources that are assumed to be more likely to contribute to the recorded data. There was no constraint posited on the dipole orientation (we used free orientation), the regularization constant was 1% and we did not apply any normalization (although we did use the residual variance fit criterion). Regions of interest were defined in terms of Brodmann areas (BAs), which were isolated with the Talairach Daemon from the BESA source space (see^20^ for full details on the minimum norm estimation procedure). All data files are available upon request.

### Data analysis

*Behavioral data*. In the English experiment, participants’ vocal responses were evaluated for each trial and reaction times corresponding to erroneous responses [incorrect naming, verbal disfluencies (i.e., utterance repairs, stuttering) and nonresponses] were excluded from further analysis. In the ASL experiment, all signs were video recorded and trials containing incorrect signs or disfluencies were rejected from behavioral analyses. Trials containing reaction times below or above 2.5 SD from the mean were also discarded. Reaction times were averaged over trials per condition and per participant and subjected to Language (English/ASL) x Composition (Phrase/List) 2 × 2 mixed design ANOVAs.

### ROI main analyses

In the statistical analysis, data for each time point were submitted to 2 × 2 cluster permutation mixed-design ANOVAs (between-subjects factor: Language (ASL/English); within-subjects factor: Composition (Phrase/List)). As the main goal of the current study was to compare combinatory effects in English and ASL, the analyses focused on the LATL (including BA38) and the vmPFC (encompassing left and right BA11) following previous studies with closely similar designs that have found composition effects in these areas^8,9,34^. Results on these ROIs were complemented by analyses of two other regions, the angular gyrus and the left inferior frontal gyrus, which have been hypothesized as participating in aspects of syntactic/semantic processing (BA39^26^; BA44 and BA45^12,27^), but have not shown sensitivity to basic composition in production, as targeted here. This made these areas useful control regions for the present investigation. Finally, in addition to analyzing these left hemisphere regions, we also conducted a separate control analysis of the right hemisphere homologues of these regions.

ROI time courses were analyzed with non-parametric permutation tests^48^ aimed at identifying clusters of time-points during which activity localized to each region differed significantly between conditions. Prior to this, MEG activity was averaged across all sources within an ROI. Time points were first grouped into clusters representing possible effects based on criteria adopted from prior studies (p < 0.3 for at least 10 adjacent time points as in^8,9,29^). For each cluster surviving these criteria, we constructed a test statistic that was equal to the summed F-values of the point-by-point test-statistics over the selected cluster interval. The data within the largest cluster were then randomly permuted 10,000 times assigning condition labels randomly within each participant’s data, and the final corrected p-value of the observed data was calculated as the ratio of permutations yielding a test statistic greater than the actual observed test statistic. Due to the last step, this test is only capable of identifying one effect (the largest effect) within any given analysis interval and thus in order to be able to characterize potential earlier and later effects, all analyses were conducted both in an early (100–300 ms) and a late (300–500 ms) time window. The standard alpha-level of *p* < 0.05 was used to determine significance, and cluster level *p*-values were corrected for multiple comparisons across all ROIs using the False Discovery Rate^49^. Incorrect trials were excluded from this analysis.

#### Representational Similarity Analysis (RSA)

For regions showing similar combinatory profiles in ASL and English in the main analysis, we additionally ran a representational similarity analysis (RSA^15^) to further assess the similarity of the computations in these regions. Specifically, the analysis compared the similarity between the actual MEG data and a model that distinguished between composition and list trials irrespective of language. In the model representational dissimilarity matrix (RDM), trials were coded as similar in terms of their compositionality (or lack thereof) irrespective of language. For the brain RDM, we averaged into a single evoked response all repetitions of the same stimulus. This allowed us to create a stable representation per stimulus type (e.g., all instances of the phrase *white lamp* were averaged together). Then, we calculated the dissimilarity between the activity patterns associated with any two stimuli. As recommended in^15^, we used correlation distance (1-correlation) as the dissimilarity measure. This dissimilarity measure was computed from the correlation between the source amplitude of all sources within an ROI across a 50 millisecond moving window (moving millisecond by millisecond). We then ran a Spearman correlation between our model RDM and our data RDM to establish a time course of rho values representing the level of similarity between our model and our MEG data at each millisecond. Significance level was set at *p* < 0.05 and corrected for multiple comparisons with FDR.

## Electronic supplementary material

Supplementary figure
