# Supplementary material for: Shared neural correlates for building phrases in signed and spoken language
Source: Sci Rep. 2018 Apr 3;8:5492. doi: 10.1038/s41598-018-23915-0 (PMC5882945; doi:10.1038/s41598-018-23915-0)
Supplement: Supplementary file 1 — Supplementary figure [file 41598_2018_23915_MOESM1_ESM.pdf]

Shared neural correlates for building phrases in signed and spoken language

Esti Blanco-Elorrieta[1,2*] Itamar Kastner[3,4], Karen Emmorey[5] and Liina Pylkkänen[1,2,3]

[1] Department of Psychology, New York University, New York, NY, USA.

[2] NYUAD Institute, New York University Abu Dhabi, Abu Dhabi, UAE.

[3] Department of Linguistics, New York University, New York, NY, USA.

[4] Institut für Anglistik und Amerikanistik, Humboldt-Universität zu Berlin, Berlin, Germany

[5] School of Speech, Language and Hearing Sciences, San Diego State University, CA, USA.

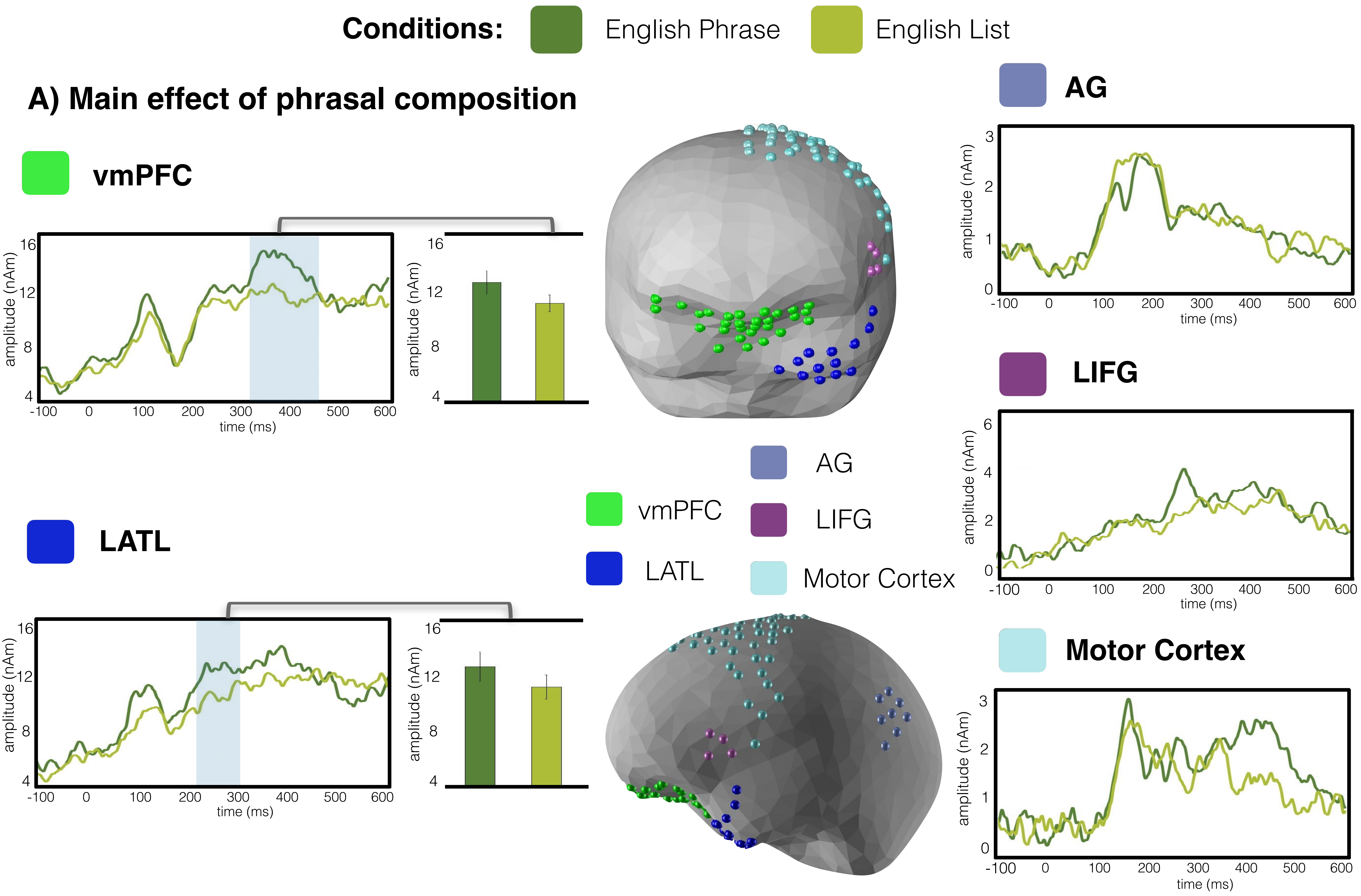
Conditions:
English Phrase
English List
A) Main effect of phrasal composition
vmPFC
amplitude (nAm)
16
12
8
4
-100
0
100
200
300
400
500
600
time (ms)
LATL
AG
LIFG
Motor Cortex
vmPFC
LATL
AG
3
2
1
0
LIFG
6
4
2
0
Motor Cortex
